# Myelin Pathology: Involvement of Molecular Chaperones and the Promise of Chaperonotherapy

**DOI:** 10.3390/brainsci9110297

**Published:** 2019-10-30

**Authors:** Federica Scalia, Antonella Marino Gammazza, Everly Conway de Macario, Alberto J. L. Macario, Francesco Cappello

**Affiliations:** 1 Department of Biomedicine, Neuroscience and Advanced Diagnostics (BIND), University of Palermo, 90127 Palermo, Italy; antonella.marino@hotmail.it (A.M.G.); francapp@hotmail.com (F.C.); 2 Euro-Mediterranean Institute of Science and Technology (IEMEST), 90139 Palermo, Italy; econwaydemacario@som.umaryland.edu (E.C.d.M.); AJLMacario@som.umaryland.edu (A.J.L.M.); 3 Department of Microbiology and Immunology, School of Medicine, University of Maryland at Baltimore-Institute of Marine and Environmental Technology (IMET), Baltimore, MD 21202, USA

**Keywords:** myelin, myelin pathology, myelinopathies, proteinopathies, chaperonopathies, Hsp60, CCT, chaperonotherapy

## Abstract

The process of axon myelination involves various proteins including molecular chaperones. Myelin alteration is a common feature in neurological diseases due to structural and functional abnormalities of one or more myelin proteins. Genetic proteinopathies may occur either in the presence of a normal chaperoning system, which is unable to assist the defective myelin protein in its folding and migration, or due to mutations in chaperone genes, leading to functional defects in assisting myelin maturation/migration. The latter are a subgroup of genetic chaperonopathies causing demyelination. In this brief review, we describe some paradigmatic examples pertaining to the chaperonins Hsp60 (HSPD1, or HSP60, or Cpn60) and CCT (chaperonin-containing TCP-1). Our aim is to make scientists and physicians aware of the possibility and advantages of classifying patients depending on the presence or absence of a chaperonopathy. In turn, this subclassification will allow the development of novel therapeutic strategies (chaperonotherapy) by using molecular chaperones as agents or targets for treatment.

## 1. Introduction

Normal physiological functioning of the nervous system depends on the presence of healthy myelin. Myelin is a complex mix of proteins and other molecules that wraps neuronal extensions such as axons, providing them with the properties necessary to ensure adequate transmission of neural impulses. Healthy myelin, in turn, depends among other factors on the normality of its protein components, and this is directly the consequence of the action of various elements, with one of the most important being the chaperoning system, which is in charge of protein quality control throughout the body, including the nervous system. The central components of the chaperoning system are the molecular chaperones, which if defective can cause diseases, the chaperonopathies [1]. A variety of molecular chaperones, on the other hand, have been demonstrated to exert therapeutic effects in various experimental models [2,3].

Since most proteins need chaperone assistance for maturation into a functional molecule, and as the chaperoning system is present in all tissues, it is likely that some chaperonopathies may contribute to diseases characterized by myelin pathology, with one or more myelin proteins qualitatively and/or quantitatively abnormal. 

Clarification of the role of chaperones in human myelin biogenesis and pathogenesis is challenging for various reasons: (1) the difficulty inherent in obtaining nervous tissue from healthy and diseased individuals; (2) the scarcity of information on which myelin protein interacts with which chaperone; and (3) the typical complexity of chaperone–client protein interplay, involving chaperoning teams and networks with participation of other interactors that vary depending on the tissue and anatomical region considered. 

Nevertheless, elucidation of the role of defective chaperones in myelin pathology is a worthwhile effort that will open the road to more accurate diagnoses and to the development of efficacious treatments focused on the sick chaperone. In this article, we will present suggestions on how it is possible to make progress in this field. For this purpose, we will discuss mutations in the genes encoding the human subgroup of chaperones called chaperonins, namely Hsp60 and CCT (chaperonin-containing TCP-1, also called TRiC for TCP-1 ring complex). Mutations in the *hsp60* gene cause SPG13, and MitCHAP-60 disease, and mutations in the gene *cct5,* encoding subunit 5 of CCT, cause a distal neuropathy [4,5,6]. These diseases are chosen as paradigms to help understand the medical problem, but the main idea is to alert scientists and physicians in practice and research to the possibility that chaperonopathies may be present in patients with myelin pathology, typically attributed to other genetic or acquired mechanisms. 

**Figure 1:**
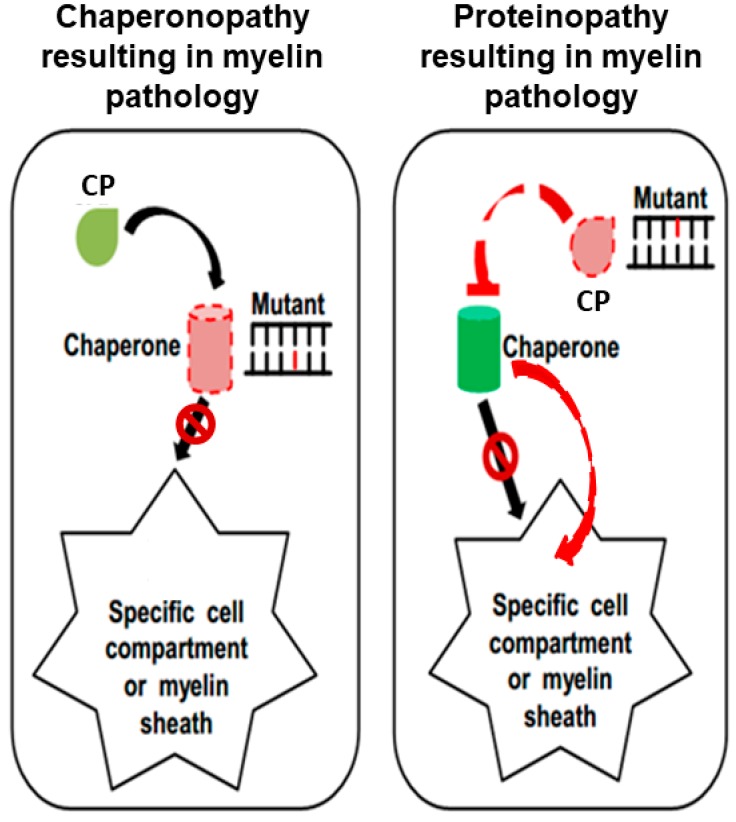
Hypothetical mechanisms involving chaperones in myelination and myelin pathology. Molecular chaperones (normal: green cylinder; abnormal: pink cylinder with dashed-red borders) assist the folding of myelin proteins (drop-shaped icons; normal, light green; abnormal, pink with dashed-red border) in the cell and their migration toward the myelin sheath, and/or directly in situ in the myelin sheath. In order to perform these tasks, chaperones bind their client proteins (substrate; client myelin polypeptide (CP)). The left panel illustrates a chaperonopathy (chaperone gene mutant, indicated by Mutant), that is, a chaperone deficiency causes incorrect myelination. An abnormal chaperone is the primary cause of myelinopathy: the chaperone’s lack of function, or partial insufficiency (e.g., due to mutation in its gene) cannot correctly fold its client myelin polypeptide (CP) and/or cannot assist its migration to its functional residence (black arrow with forbidden sign). In the right panel, the myelinopathy is caused by a mutation in the gene encoding the myelin protein affected (indicated by Mutant), while the chaperone genes are normal. An indirect chaperone insufficiency may occur if the chaperone cannot bind and interact with its substrate because the latter is abnormal, and thus it cannot be recognized or bound properly by the chaperone (top red truncated arrow); or the normal chaperone can bind the defective protein substrate but cannot fold and transport it to the place in which it functions, for example, the myelin sheath (black arrow with forbidden sign); or the chaperone transports the client protein to the pertinent compartment, but the protein does not correctly function due to mutation (curved red arrow to the right).

Genetic myelinopathies can, in principle, be due to a mutation in a gene encoding a myelin protein, in which case we have a proteinopathy, and the involvement of molecular chaperones in the mechanism of disease may not be the most determinant (Figure 1, right panel). In contrast, there might be myelinopathies that are directly associated with mutation of a chaperone gene, whose protein product (i.e., the molecular chaperone) is defective and unable to assist in the production and transport of one or more myelin proteins (Figure 1, left panel). In this case, we have a chaperonopathy that contributes to the mechanism of myelinopathy.

Whether Hsp60 and CCT have myelin proteins as clients is not yet clear, however it has been shown that both chaperonins interact with members of the Hsp70 family, such as Hsc70 (HSPA8) and the stress-inducible Hsp70 (HSPA1A), which in turn have the following pertinent characteristics: (1) they are part of the myelin proteome [7,8]; (2) they participate in the folding and transposition of the myelin basic protein, which constitutes 30% of the myelin proteins in the central nervous system [7]; and (3) they collaborate with Hsp60 and CCT in the maintenance of protein homeostasis [9]. To date, mutations in a member of the Hsp70 family linked to neurodegenerative and myelin diseases are very rare, for instance a mutation on an Hsp70 nuclear transporter, the Hikeshi protein, which causes a congenital leukodystrophy, has been described [10]. The disease was discovered in Jewish Ashkenazi families and is characterized by early onset microcephaly, optic atrophy, and spastic paraparesis.

The examples chosen for discussion here can be classified into two categories: (a) the hereditary spastic paraplegia (HSP) together with the hypomyelinating leukodystrophy (HLD) associated with mutations in the gene encoding the chaperonin of Group I, Hsp60, namely SPG13 and MitCHAP-60 (or hypomyelinating leukodystrophy 4 (HLD4) (OMIM **#**612233)) disease [4]; and (b) the distal sensory neuropathy associated with a mutation in the gene encoding one of the eight subunits of the Chaperonin of Group II, CCT [5,6].

In MitCHAP-60 disease and in the distal neuropathy caused by a CCT5 mutation, there is indication of myelin damage, but no definitive evidence of myelin abnormality has been reported for the SPG13 cases discussed here. It is, however, noteworthy that the penetrance of the autosomal dominant SPG13 is age-dependent and incomplete, therefore, the clinical-pathological features vary among patients, with some showing the signs mentioned in Table 1, while others do not.

Even if in any given patient the results of the neurological examination appear nearly normal, the disease SPG13 cannot be ruled out because the pathogenic process may not yet be fully developed at the time of the clinical examination. Therefore, the question of whether myelin pathology is a pathogenic factor in SPG13 remains open for investigation, and this is one of the reasons we have included this disorder for discussion here. Furthermore, the hereditary spastic paraplegia 2 (SPG2), the prototype of early-onset hypomyelinating leukodystrophy Pelizaeus–Merzbacher Disease (PMD) (MIM 312080), and the hereditary spastic paraplegia 35 (SPG35), have been associated with mutations in the *PLP1* (SPG2 and PMD) and *FA2H* (SPG35) genes, both genes coding for important components of myelin sheath [15,16]. It is therefore clear that proteinopathies are pathogenic factors, if not the pathogenic factor, of these diseases. This immediately directs the attention to the chaperoning system and its role in protein homeostasis and the probability of its participation in the disease mechanism, if nothing else because of the loss of its interaction with the defective myelin protein that is no longer recognizable by the pertinent chaperone. 

## 2. The Structure of Hsp60 and CCT Chaperonins 

The chaperonins are a subgroup of chaperones characterized by a molecular weight close to 60 kDa that form ring-shaped oligomers, which in turn associate end-to-end to build a double-ring structure with a central cavity, inside which polypeptides are folded into mature proteins with native conformation. Hsp60 form homo-heptameric rings, whereas CCT subunits form hetero-octameric rings, and consequently, the fully functional double-ring chaperoning complex is a tetradecamer for Hsp60 and a hexadecamer for CCT. When the Hsp60 molecule or one subunit of CCT is mutated, the corresponding chaperoning oligomer may be defective in its stability and chaperoning ability, which results in disease, as illustrated by the examples discussed below.

## 3. Mutations in the *hsp60* Gene 

Mutations in the *hsp60* gene have been found associated with a phenotype matching that of the primary hypomyelinating leukodystrophies (HLD) [4], Table 1. Its protein product, the mitochondrial chaperonin Hsp60, participates in the folding and assembly of newly synthesized polypeptides inside the mitochondria [17].

The Hsp60-associated diseases discussed here are the mitochondrial chaperonopathies MitCHAP-60 (HLD4) disease and the hereditary spastic paraplegia (HSP) SPG13 [4,12]. A summary of the main genetic, clinical, and pathological features of HLD and HSP is presented in Table 1. HSP belongs to the group of the upper motor neuron distal neuropathies and is a heterogeneous disease, or perhaps a group of diseases, with an age of onset typically in adulthood [18], for which about 60 different causative genes have been identified, but others may still be discovered [19]. It is likely that some of these “other” genes to be discovered will be chaperone genes. This is the main point of this article, namely, to prompt the discovery of new gene variants causing myelinopathies, including a search for variants in genes encoding chaperones. 

It has been hypothesized that the mutations in MitCHAP-60 and SPG13, cause a protein transport failure affecting the conveyance of myelin-forming proteins along the axons, and also cause mitochondrial malfunction [12,20,21]. Axons and mitochondria are dependent on each other: the mitochondria travel along the axon using its transport system, and this system needs energy produced by the mitochondria for efficient transport, thus when one fails the other also suffers. 

SPG13 was associated with an autosomal dominant mutation (c.292G > C; p.Val98Ile) that caused disease in heterozygous individuals of a French family [11,22,23]. To investigate the molecular mechanisms affected, the mutant protein was analyzed in vitro and in vivo using an engineered *Escherichia coli* [24]. It was observed that when the chaperonin tetradecamers consisted of only mutant subunits, the ATPase activity was seriously compromised and so was substrate folding. However, when the chaperonin tetradecamer was formed by subunits with the Val98Ile mutation mixed with Hsp60 wild-type subunits, no predominant negative effect by the mutation was observed: the chaperoning activity was still present, albeit reduced. 

Another heterozygous missense mutation, p.Gln461Glu, in the *hsp60* gene in one out of 23 Danish SPG patients has been identified. Due to low penetrance, it can be asymptomatic or symptomatic (Table 1), and the extent of pathogenicity of this mutation has not been established [13]. By means of a complementation assay, it was established that *E. coli* cells expressing Hsp60-p.Gln461Glu show a mild functional impairment, therefore, this variant may be disease-associated with low penetrance. It is possible that the Gln461Glu missense mutation does not cause SPG by itself, but instead causes SPG when it is combined with other genetic or environmental risk factors. Even if the mutant Val98Ile was shown to be functionally more severely impaired, studies with *E. coli* GroEL, indicated that both amino acid variants, p.Gln461Glu and p.Val98Ile, are likely to affect the same functional domain of the Hsp60 molecule. These mutations appear to interfere with the correct conformational change required for ATP binding. Also, bioinformatics analysis predicted detrimental molecular effects for the Hsp60-p.Val98Ile and Hsp60-Gln461Glu variants, but no marked effects for the Hsp60-p.Asp29Gly, although this variant is implicated in causing disease [25,26]. In the diseases associated with Hsp60-p.Val98Ile and Hsp60-Gln461Glu variants, myelin impairment has not been confirmed or excluded, and this issue remains open for investigation.

The Hsp60-p.Asp29Gly missense mutation (g.1512A/G on 2q32.3-q33.1 locus) was described as causing an autosomal-recessive hypomyelinating leukodystrophy that was designated MitCHAP-60 disease or HLD4, or a “complicated” SPG [12]. The ten patients studied presented myelin defect and the cardinal features reported in Table 1.

The proposal that the Asp29Gly mutation was the cause of MitCHAP-60 disease was backed up by several findings: (1) it was observed that the phenotype and the mutation consistently co-segregated in the suspected members of the same family; (2) this same mutation was not found in healthy control individuals of the same ethnic group; (3) the Asp29 is strongly conserved within the Hsp60, from bacteria to humans, suggesting indispensability of this amino acid; (4) the *E. coli* complementation tests showed that the mutated homologous Hsp60 fails to support the bacterium’s survival, especially at high temperatures [12]; and (5), in vivo studies demonstrated a myelin dysfunction in corpus callosum of transgenic mice harboring the mutation [13]. 

Of great interest are the mechanisms operating in the mutations of Hsp60 that can lead to such substantial differences, both in the mode of inheritance and in the phenotype of the diseases SPG13 and MitCHAP-60. The Asp29Gly mutation has no apparent pathogenic effect in heterozygosis, but it is pathogenic in homozygosis, although still allowing partial Hsp60 functionality. Instead, the Val98Ile mutation is pathogenic in heterozygosis, causing a reduced functioning of the Hsp60. It is noteworthy that the Asp29Gly mutation causes a more severe and precocious disease than the Val98Ile mutation, even if the latter has more damaging effects on Hsp60 functions [12].

More recently, a new case of MitCHAP-60 with the homozygous mutation Asp29Gly has been reported [14]. The patient was a two-year-old Syrian boy and showed abnormal myelination and other signs and symptoms mentioned in Table 1.

There is insufficient information on the possible interactions of Hsp60 and myelin proteins. Only few studies on the morphology and mitochondrial dynamics following expression of the mutant forms associated with SPG13 and MitCHAP-60 have been performed in Cos-7 cells [20,21]. The Hsp60-p.Asp29Gly, or Hsp60-p.Val98Ile, or Hsp60-p.Gln461Glu mutation caused a rise in the number of mitochondria, the appearance of short mitochondria, and a mitochondrial membrane decrease, whereas cells expressing the Hsp60 wild type did not show these abnormalities. 

## 4. Mutation in the CCT5 Subunit Gene 

Another disease characterized by hypomyelination in the posterior tract of the spinal cord is caused by the mutation His147Arg in the *cct5* gene, encoding the subunit 5 of CCT complex, with clinical and pathological manifestations characteristic of a distant sensory neuropathy (Table 1) [5,6]. The His147Arg homozygous mutation was observed in all four patients studied. The amino acid change affects the equatorial domain of the CCT5 subunit, with predictable defects in ATP binding and hydrolysis and chaperoning ability. Studies with an archaeal model showed that the His147Arg mutation impairs ATPase activity and the ability to form stable hexadecamers, which results in impairment of chaperoning ability [27]. Similar defects were also found using recombinant human CCT5 [28,29]. Likewise, it has been shown that rats with mutated *cct4* gene have a similar disease phenotype to that observed in humans with the *cct5* mutation discussed above [30]. 

It is pertinent to mention that while CCT interacts mainly with actin and tubulin to assist in their correct folding, this chaperonin also interacts with 5%–10% of the mammalian proteome [31]. Therefore, it is possible that mutations in CCT subunits disturb the transport of myelin proteins on the cytoskeleton, or their correct folding, if they happen to be CCT clients that still have to be identified.

## 5. Chaperonotherapy

Chaperonopathies can be classified according to various criteria like any other disease; for example, chaperonopathies can be genetic or acquired, with the former caused by pathogenic variants of a chaperone gene, as discussed above [1]. Acquired chaperonopathies are those in which chaperones are quantitatively and/or qualitatively abnormal but chaperone genes are normal. Another classification of chaperonopathies is based on molecular mechanisms and includes the chaperonopathies by defect, by excess, and by mistake, Table 2 [32]. 

**Figure 2:**
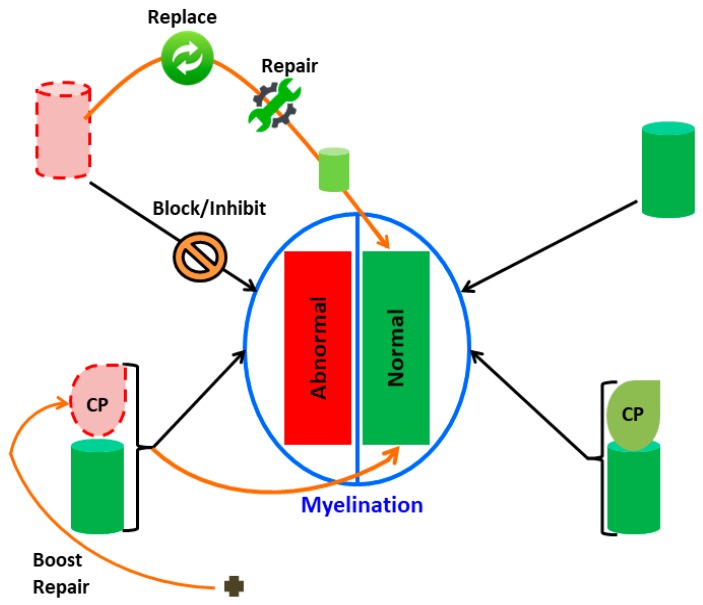
Possible pathways to investigate the mechanisms of myelination defects involving chaperones and pertinent chaperonotherapy strategies. Normal and abnormal myelination (green and red rectangles, respectively) are shown within the central oval. Chaperones (cylinders) normal (green) or abnormal (pink with red-dashed borders); pertinent client proteins (CP; drop-shaped icons) may be normal (green) or abnormal (pink with red-dashed borders). The normal mechanism of myelination is represented on the right, while mechanisms involved in abnormal myelination are schematized on the left side of the figure; there are here two possibilities: chaperone failure (top) or client polypeptide (CP) defect (bottom). Chaperone failure is amenable to positive chaperonotherapy consisting of chaperone replacement (gene or protein, or artificial chaperone) or chaperone repair (by means of chaperone-activating compounds). Also shown is the case of a chaperone interfering with the normal process of myelination, that is, chaperonopathy by mistake, which is amenable to negative chaperonotherapy, consisting of blocking/inhibiting the pathogenic chaperone, as shown by the descending black arrow crossed with a forbidden sign. If the client protein is defective (proteinopathy; bottom), it is possible to boost it with chemical chaperones (black cross at the bottom) and reconstitute its function, at least partially.

For each type of chaperonopathy, one can think of an appropriate modality of treatment centered on the affected chaperone. Positive chaperonotherapy encompasses the replacement of a defective chaperone, applying gene therapy or administering the normal version of the abnormal chaperone protein. On the other hand, negative chaperonotherapy is required to treat a chaperonopathy in which the abnormal chaperone plays an active role in the mechanism of disease, for instance in some cancers in which a chaperone is essential for cancer-cell growth, proliferation, and metastasization. Examples of these two modalities of chaperonotherapy are schematized in Figure 2. 

Results from in vitro and in vivo studies indicate that chaperones can modulate and slow down neurodegeneration and are, therefore, promising therapeutic agents for neurodegenerative disorders [34,35]. Likewise, enhancement of chaperone pathways by inhibiting Hsp90 was beneficial for myelin formation by Schwann cells from neuropathic mice with abnormal expression of PMP22 [36]. PMP22 abnormal expression is associated with Charcot–Marie–Tooth disease type 1A demyelinating neuropathy [37,38,39,40]. The results indicated that non-myelinating and myelinating glial cells respond to EC137 (a small molecule inhibitor of Hsp90) by increased expression of chaperones, including Hsp70, Hsp27, and αB-crystallin. Noteworthy, the enhancement of these chaperones was associated with a pronounced improvement in myelination in neuron–glia explant cultures from neuropathic mice, as compared with untreated controls.

The molecular processes that underlie the functions of Hsp60 and CCT5, wild-type or mutated, are not yet fully known. Regarding the neurodegenerative chaperonopathies reported here, it would be appropriate to think of positive chaperonotherapy, aiming to recover/improve the functional abilities of the defective chaperonins Hsp60 and CCT. 

Several compounds have been evaluated in recent years to induce the cytoprotective ability of heat shock proteins. For instance, the antibiotic geldanamycin, natural compounds, and bioactive molecules derived from plants act as stress proteins activators [41].

Since Hsp60 is often increased and operating in favor of pathology, compounds able to inhibit Hsp60 have been studied [42]. Not much is known about chemicals or molecules effective at inducing production of intracellular Hsp60. In this regard, it has been reported that cytokines can increase the expression of Hsp60 in adult human astrocytes [43].

CCT is also increased in a variety of tumors such as lung cancer [44], so several CCT inhibitors are being studied as possible therapeutic agents. On the other hand, over the past two years it has been demonstrated that the CCT5 subunit has an important role in promoting axonal transport in neurodegenerative diseases [45,46]. The identification of compounds capable of inducing CCT expression is still a little-explored field and a complicated one due to the fact that multiple intracellular pathways, in which CCT is involved, must be maintained within a physiological balance that cannot be much modified with foreign agents.

## 6. Conclusions and Perspectives for the Future

The take-home message intended to convey with this short article is that a search for chaperonopathies in neurological disorders with myelin pathology promises to unveil new disease mechanisms involving abnormal chaperones. This investigative approach should be applied not only in patients in whom causative gene variants affecting non-chaperone genes have been identified, but also in patients in whom causative genes have not been identified but clinical data suggest a myelin impairment. In this way, it will be possible to tell apart patients with very similar clinicopathological–biochemical features but differing in the functioning of their chaperoning system. If a chaperonopathy is detected, then the therapeutic opportunities expand and chaperonotherapy may come to the rescue.

## Figures and Tables

**Table 1:** Diseases with myelin abnormality associated with chaperone gene mutations.

Disease Myelin Status Chaperonopathy	Mutation	Genetic Condition	Clinical-Pathological Features	Reference
Peripheral neuropathy Demyelinating By defect	CCT5-p.His147Arg (consanguineous Moroccan family)	HET	Progressive distal sensory neuropathy of upper and lower limbs leading to mutilating acropathy; abnormality of the lipoprotein profile; severe atrophy of the spinal cord predominantly in the posterior tract (MRI).	[6]
SPG13 Possibly demyelinating By defect	Hsp60-p.Val98Ile (French family)	HET ^1^	Severe functional handicap; decreased vibration sense; urinary urgency; pes cavus; increased reflexes in the lower and upper limbs; loss of Babinski sign.	[11]
MitCHAP-60 disease Hypomyelinating By defect	Hsp60-p.Asp29Gly (consanguineous Israeli Bedouin kindred)	HOM	Rotatory nystagmus, progressive spastic paraplegia; variable rate of neurological deterioration and regression; severe motor impairment; abnormal head control; profound mental retardation; hypomyelinating leukodystrophy (MRI).	[12]
Asymptomatic or symptomatic	Hsp60-p.Gln461Glu (Danish HSP patients)	HET	Asymptomatic or symptomatic. Symptomatic cases show: spasticity and weakness in the lower limbs and impaired vibration sense in the toes; normal cerebrum and spinal cord MRI; abnormal motor-evoked and somatosensory evoked potentials; evoked potentials (VEP) abnormal on the left eye.	[13]
MitCHAP-60 disease Hypomyelinating By defect	Hsp60-p.Asp29Gly (Syrian boy)	HOM	Slow psychomotor development; absence of heat control; hypotonia; nystagmus; limb spasticity; feeding difficulties; no evidence of normal myelination (MRI)	[14]

^1^ Abbreviations: HET, heterozygosity; HOM: homozygosity; MRI, magnetic resonance imaging; HSP: hereditary spastic paraplegia. Note: the list of myelin disorders primarily or secondarily dependent on chaperones that are abnormal due to genetic or acquired defects will most likely increase quite significantly in the near future, if clinicians and pathologists are aware of their existence and look for them.

**Table 2:** Chaperonopathies grouped according to the type of chaperone abnormality and pertinent chaperonotherapy modality.

Chaperonopathy by:	Mechanism, Features	Chaperonotherapy Mode
Excess	Quantitative, e.g., due to gene dysregulation; upregulation; other.	Negative: Chaperone gene knockdown; inhibition by miRNAs; chaperone-protein blocking (compounds)
	Qualitative, e.g., gain of function.	Negative: Chaperone gene knockdown; inhibition by miRNAs; chaperone-protein blocking (compounds).
Defect	Quantitative, e.g., gene downregulation; absence or misplacement; sequestration; excessive demand (defect relative to substrate availability); other.	Positive: Chaperone gene/protein replacement; artificial chaperones; chaperone gene induction (e.g., mild harmless stressors); combined.
	Qualitative, e.g., due to structural defect genetic or acquired (e.g., aberrant post-translational modifications).	Positive: Chaperone gene/protein replacement; artificial chaperones; chaperone function boost (compounds); combined.
Mistake	Normal chaperones (at least as far it can be determined with current methodologies) contribute to disease, e.g., tumors that need chaperones to grow; autoimmune conditions in which a chaperone is the autoantigen and/or induces production of pro-inflammatory cytokines.	Negative: Chaperone gene knockdown; inhibition by miRNAs; chaperone-protein blocking (compounds); combined.

Modified and updated from [33].
